# Hypertension and Cardiovascular Risk Among Children with Chronic Kidney Disease

**DOI:** 10.1007/s11906-024-01308-1

**Published:** 2024-05-28

**Authors:** Nicholas G. Larkins, Jonathan C. Craig

**Affiliations:** 1 Department of Nephrology and Hypertension, Perth Children’s Hospital, Nedlands, Australia; 2 Medical School, University of Western Australia, Perth, Australia; 3 College of Medicine and Public Health, Flinders University, Adelaide, Australia

**Keywords:** Hypertension, Child, Infant, Adolescent, Cardiovascular risk, Renal insufficiency, Chronic

## Abstract

**Purpose of Review:**

Cardiovascular disease is the most common cause of mortality across the lifespan of children with chronic kidney disease (CKD). Hypertension is a common and important contributor, but other factors such as obesity, dyslipidemia and mineral bone disease play a role. This narrative review focusses on studies published in the past five years that have investigated hypertension and cardiovascular risk among children with CKD.

**Recent Findings:**

Cohort studies such as Chronic Kidney Disease in Children (CKiD) and Cardiovascular Comorbidity in Children with CKD (4C) have continued to develop our understanding of blood pressure (BP) phenotypes, and of progressive changes in the structure and function of the heart and blood vessels occurring in children with CKD. Metabolic risk factors, such as dyslipidemia, may represent an under-recognized component of care. Trial data are less common than observational evidence, but support lifestyle interventions currently used, mainly the low sodium dietary approaches to stop hypertension (DASH) diet. The findings of the recently reported Hypertension Optimal Treatment in Children with Chronic Kidney Disease trial (HOT-KID) are described in relation to the use of office BP treatment targets.

**Summary:**

Cardiovascular health is critical to the long-term outcomes of children with CKD. Recognizing and treating hypertension remains a critical component to improving outcomes, along with measures to improve concurrent cardiovascular risk factors. Some cardiovascular changes may not be reversible with transplantation and further research is needed for children at all stages of CKD.

## Introduction

The kidneys play an integral role in maintaining normal blood pressure via salt and water homeostasis. Abnormalities in kidney function are thus commonly associated with abnormalities in blood pressure. In young children with congenital anomalies this may be low blood pressure due to salt wasting, but more commonly, and with disease progression, hypertension due to hyperreninaemia and salt retention prevails. Hypertension itself has negative impacts on the glomerulus and hastens progression of chronic kidney disease (CKD). Cardiovascular disease is the most common cause of death children with kidney failure, accounting for about one-third of all deaths [1, 2].

This review focuses on studies published within the last 5-years and includes summaries of more than 40 manuscripts. This expanding body of research is encouraging as we attempt to unravel the relationship between hypertension, CKD, and cardiovascular risk factors, to improve long-term outcomes for these at-risk patients.

## Prevalence and Impact of Hypertension Among Children with CKD

The largest longitudinal cohorts for children with CKD are the Chronic Kidney Disease in Children (CKiD) and Cardiovascular Comorbidity in Children with CKD (4C) studies, conducted in North America and Europe, respectively [3, 4]. Both studies have consistently identified a prevalence of hypertension among children with CKD of between 25 and 50%, the higher end of this range applying to those with CKD stage 5 [4, 5]. This compares to a prevalence of hypertension of 4% among all children [6–8].

The prevalence of hypertension among children with CKD does not vary appreciably with age. However, in CKiD, younger children were significantly less likely to have well-controlled hypertension (25% of children < 7 years compared to 39% among those ≥13 years) [9]. In that study, children < 7 years were half as likely to receive pharmacotherapy for hypertension compared to children ≥13 years.

The CKiD study also examined temporal trends in blood pressure classification by comparing participants’ clinic and 24-h ambulatory blood pressure monitor (ABPM) values enrolled during two time periods, 2005 to 2008 and 2010 to 2013 [10]. While there was a significant improvement in the unadjusted clinic BP values, this was entirely accounted for by differences in the CKD classification (etiology, glomerular filtration rate, proteinuria) and other known confounders such as socioeconomic status. The ABPM data indicated that despite a lower proportion of participants having confirmed hypertension in the latter period, there was no improvement in blood pressure control overall due to a concurrent increase in masked hypertension. Rather, the proportion with normotension according to ABPM fell from 42 to 37%.

The impact of hypertension among children with kidney failure warrants specific discussion. Current United States (US) Renal Data System (USRDS) data indicates that 39% of deaths in people younger than 30 years of age with kidney failure are due to cardiovascular causes, with a five-year cumulative incidence of cardiovascular death ranging from 4% among children (1 to 11 years old) to 7% among young adults (22 to 29 years old) [11]. These findings are consistent with European data confirming cardiovascular events as the most common cause of death among children with kidney failure [1]. Cardiovascular death is most common among people initiated on hemodialysis, although the hazard is not proportional and after two years is similar to those receiving peritoneal dialysis [12]. There are data to suggest an improvement in cardiovascular mortality during the past two decades, which would be consistent with improvements in overall mortality among children with kidney failure [13]. However, limitations in registry data such as misclassification bias and missing data reduce the certainty about these conclusions [1]. Correctly classifying cause of death is difficult among people with kidney failure because cardiovascular death may occur due to arrythmias provoked by electrolyte disturbances, accelerated atherosclerosis, and/or cardiac failure. Studies conducted in adults indicate that hyperkalemia is likely to be the most common cause, based on the timing of cardiovascular events in relation to dialysis sessions and frequency of bradycardic rhythms pre-arrest [14, 15]. What remains to be clearly understood is the relative contribution of pre-existing atherosclerotic, cardiac, and other vascular changes. For those with a functioning kidney transplant, the risk of cardiovascular death is less, but remains markedly higher than for age-matched peers [16].

Hypertension has adverse effects beyond the cardiovascular system directly, with hypertension associated with a greater rate of decline in GFR among children with CKD [4]. In 11240 children followed for a median of 5.1 years (interquartile range [IQR] 2.8 to 8.3), hypertension with or without proteinuria was found to be independently associated with the development of CKD 5 or a halving of GFR (adjusted hazard ratios 1.49 [95% CI 1.22 to 1.82] and 3.98 [95% CI 3.4 to 4.68], respectively) [17]. These findings are similar to data from a study that quantified the impact of hypertension among children with congenital anomalies followed from early in life (adjusted odds ratio for development of CKD 1.6, 95% CI 0.7 to 3.6) [18].

There is increasing evidence to support an association between hypertension and neurocognitive outcomes. An inverse correlation between blood pressure and performance on neurocognitive testing has been demonstrated for children with and without CKD [19, 20]. There are now also longitudinal data from the Young Finns Study demonstrating an association between childhood BP and cognitive function in mid-adult life in the general population (0.42 SD difference in paired-associates learning test between extreme quartiles of childhood BP, equivalent to 8.2 years difference in cognitive age) [21]. It appears that hypertension likely forms part of the overall milieu leading to worse cognitive and academic outcomes among children with CKD [22]. Beyond the single-visit or intermittent APBM values, BP variability may contribute to or potentiate the adverse effects of hypertension. This has been examined in the CKiD study, with visit-to-visit systolic BP variability, and not short-term variability or ABPM, predictive of worse neurocognitive test performance [23]. No association between BP variability and test score trajectory over the follow-up period was observed (median 4.0 years). One difficulty in interpreting studies using outcome data derived from complex neurocognitive testing is that multiple tools may be used, with multiple domains and sub-domains assessed, resulting in large numbers of hypotheses being tested within each study, with greater multiplicity again across the totality of the evidence. A pre-defined statistical analysis plan is not always done and/or presented and it is unclear if there is a hierarchy of relevant outcomes. While some of these difficulties are unavoidable, it is important to consider the impact of multiplicity when interpreting p-values reported for individual comparisons, which leads to underestimation of the true risk of type 1 error.

## The Role of ABPM

ABPM plays an important role in the investigation and management of hypertension. The central role of ABPM in the Effect of Strict Blood Pressure Control and Angiotensin Converting Enzyme Inhibition on the Progression of Chronic Renal Failure in Pediatric Patients (ESCAPE) trial methods has been a key driver in the clinical uptake of ABPM in children. In ESCAPE the ABPM ΔBP values were also more consistent with the expected Gaussian distribution, compared to office ΔBP values, which would fit with ABPM being a more accurate measurement (total variance was also reduced, which has implications for clinical trial design) [24]. The importance of ABPM is highlighted by both the American Academy of Pediatrics (AAP) and European Society of Hypertension (ESH) Guidelines [7, 8]. Previously the two guidelines were discordant regarding the inclusion of BP load in the definition of hypertension by ABPM. However, the 2022 AAP ABPM update has removed load criteria [25, 26]. This change was based on data from CKiD and Study of Hypertension in Pediatrics, Adult Hypertension Onset in Youth (SHIP-AHOY) which demonstrated that isolated BP load elevation is a poor predictor of LVH and time to kidney failure, and that the addition of BP load to mean BP value thresholds adds little to discrimination for these events [27, 28]. This change is important because isolated BP load elevation is common among children with CKD (23% of participants in CKiD) [27]. There remain some differences between the AAP and ESH ABPM criteria in the use of thresholds for adolescents. The AAP recommends fixed thresholds from 13 years of age, compared to the ESH which uses adult values from 16 years of age. Recent data confirm that the inclusion of percentile values in adolescent definitions improves accuracy to detect LVH among children with CKD; although the clinical relevance of the modest difference observed is uncertain [29].

There are two main functions of ABPM. Firstly, the detection of children with masked or white coat hypertension who would otherwise fail to receive appropriate treatment. Secondly, to phenotype changes in BP over the course of the circadian cycle in those with known hypertension. Masked hypertension can be detected in about 25% of children with CKD, and although prevalence estimates vary, masked hypertension is more common among children with CKD than the general pediatric population and most other subgroups [30]. Masked hypertension has been shown to correlate with left ventricular hypertrophy (LVH) among children with CKD and the general pediatric population [31, 32]. Regarding the relevance of phenotyping BP among children with known hypertension, nocturnal hypertension is a better predictor of most relevant outcomes [33]. These observations extend to children with CKD, among whom nocturnal hypertension is equally as important as daytime hypertension, and together they have an additive effect on progression to kidney failure [34]. Similar findings have been demonstrated for the correlation between nocturnal hypertension and cardiovascular changes in children with CKD measured by LVH, pulse wave velocity [PWV], and carotid intima-media thickness [CIMT]) [35]. Nocturnal BP may also be expressed as a fraction of daytime BP, producing the nocturnal dipping metric. However, current data are conflicting regarding any association between dipping and end-organ outcomes among children with CKD [36–39]. Despite the benefits of ABPM, these must be balanced again the burden of collection on patients and financial cost associated with measurement. Attempts to distinguish rules that identify children with CKD at low-risk of hypertension, and thus avoid unnecessary ABPM have had limited success. Among CKiD participants with normal clinic BP, 9% with a clinic systolic BP < 20th percentile and diastolic < 80th percentile had masked hypertension [40]. This group, for whom less frequent ABPM may be appropriate, comprised 21% of the total cohort (167 of 809 children) [40].

With increasing use of ABPM, questions are now being asked about its reproducibility and the number or frequency of measurements required for accurate diagnosis. Some studies have attempted to determine reproducibility using measurements taken at distant time points, but it is not possible to distinguish measurement error from changes in the true value over time [41]. In addition, there is also substantial short-term physiological variability, for example reflected as day-to-day variability. Attempting to quantify this, meta-analysis of short-term studies among adult populations indicate that while the average difference in BP values between repeated measurements is small (pooled 24-h systolic average difference 0.71 mmHg, 95% CI -0.08 to 1.51), there is substantial variability within individuals (pooled 24-h systolic 95% limits of agreement -14.2 to 14.7 mmHg) [42]. Recent pediatric data confirm that ABPM has a lower population-level variance compared to clinic BP (important for sample size determination in clinic trials) and has good reproducibility (24-h systolic correlation coefficient 0.87). However, for comparison, the reproducibility of clinic BP in this study was also high (systolic correlation coefficient 0.81), noting that casual BP measurement in routine clinical practice is infrequently performed with the same rigor as in research environments [43, 44]. Taken together, these data suggest a need for further research into ABPM reproducibility in children, to determine the number and frequency of measurements required for accurate decision making. Such research also needs to consider the role of ABPM in relation to other less resource intensive methods that might act to reduce the number of 24-h measurements required (e.g. home BP).

## Early Cardiovascular Phenotypes

An increasing number of studies have aimed to define the importance of other cardiovascular risk factors in addition to BP. Given the long lead time to events, study outcomes are mostly assessed via surrogates related to the structure or function of the heart and vascular system.

The 4C study included 688 children with CKD stages 3 to 5, who underwent annual echocardiography, measurement of CIMT and PWV [3]. 4C has demonstrated a substantially increased prevalence of LVH among children with CKD 5 (48%) compared to CKD 3a (11%) [5]. In this study, CIMT and PWV were less closely related to eGFR, with 20% of participants having an elevated PWV, and 42% an elevated CIMT, regardless of CKD stage. These findings indicate that BP plays a greater relative role in the development of LVH than vascular abnormalities, which may be influenced by a broader array of cardiovascular risk factors. Longitudinal data investigating the progression of CIMT and PWV when on different forms of kidney replacement therapy (KRT) found a small increase in CIMT with time on dialysis (β 0.0053 mm/year, SE 0.0018), compared to stable measurements following transplantation (the non-linear hazards model estimated a reduction in CIMT several years post-transplant, but this is less certain due to the relative sparseness of observations at the latter timepoints) [45•]. The lack of significant improvement in CIMT following transplantation is important, with all models indicating that the last CIMT prior to KRT was the strongest predictor of subsequent measurements. That is, abnormalities in CIMT accrued prior to KRT may be hard to reverse even with successful transplantation. Another interesting finding from this study was confirmation of an inverse association between body mass index (BMI) and CIMT. Similar to adult patients, a low-normal BMI was a strong predictor of adverse outcomes across multiple domains [45•, 46, 47]. Regarding PWV among children progressing to KRT, and in contrast to CIMT, 4C has demonstrated continued increases in PWV after transplantation which were greater among girls than boys [48]. A separate longitudinal study, examining PWV and CKD progression (defined as 50% eGFR loss, eGFR < 10 ml/min per 1.73 m2, or the start of KRT) among children not on dialysis, failed to find an association between these variables, noting the median follow-up time was relatively short (2.7 years, IQR, 0.7 to 4.4 years) [49].

These emerging data from 4C broadly mirrors that of other studies, including a recent comprehensive assessment of 79 children and 21 young adults with CKD 4 + in the United Kingdom (UK) [50]. LVMI was higher among those on dialysis, with 27% of dialysis participants exceeding the 95th reference percentile compared to 5% of non-dialysis participants. In contrast to LVMI, functional cardiovascular abnormalities were equally common among non-dialysis patients, leading to most participants having at least one marker of an abnormal cardiac or vascular phenotype; 69.5% among those not on dialysis, 88.3% among the 77 participants on dialysis. Measures included in the composite outcome were CIMT, presence of coronary artery calcification [CAC], LVMI, vascular distensibility, PWV. The inclusion of CAC in a cohort of this size is relatively novel. While the 12% prevalence of increased CAC among participants on dialysis was less than in previous data, it is still concerning among a cohort with a median age of 14 years given the strong correlation with future CV events and high prevalence of increased CAC among adult survivors of childhood-onset kidney failure [51–53]. Contemporaneous data from Finland, examining the cardiovascular health of 51 participants with a median age of 23.5 years (IQR 9.5 to 27.8) who had received a kidney transplant in childhood, are consistent. BP was again the strongest predictor of LVMI. An elevated CAC was observed in 10% of participants and was positively associated with dialysis vintage and higher values of parathyroid hormone (PTH) during dialysis [54]. The relationship of CAC to dialysis vintage and PTH are consistent with most data (using peak or averaged PTH values) and ex-vivo models of vascular calcification in children [50–52, 55]. CIMT has also been repeatedly associated with PTH among children on dialysis; but the best predictor of PWV is less clear, with studies identifying different CKD mineral bone disease biomarkers (e.g. serum calcium, phosphate, calcium x phosphate product) as being relatively more important [51, 52].

The cardiac measures described (LVMI, LVH, CAC) mostly relate to structure as opposed to function. Previous research has not demonstrated increased functional cardiac abnormalities among children with CKD, in contrast to measures of vascular function such as PWV and distensibility. This seems incongruous with the long-term cardiovascular risk profile of the cohort. Hence, recent research has focused on the relationship between CKD and more sensitive markers of cardiac function, including novel echocardiographic measures and cardiac magnetic resonance imaging. The 4C and the Hypertension Optimal Treatment in Children With Chronic Kidney Disease Study (HOT-KID) studies recently combined data investigating the first-phase ejection fraction (EF1) as a marker of early systolic function among 321 children with CKD (stages 1 to 5, not on dialysis) compared to 63 controls [56]. These demonstrated a significant reduction in EF1 among children with CKD and a positive correlation between eGFR and EF1. Similar relationships, but with a smaller effect size, were observed for diastolic function. In contrast, there was no difference in total ejection fraction between groups. Another recent study, using cardiac magnetic resonance imaging (MRI) data indicated a clear difference in LV end-diastolic volume and ejection fraction between children with CKD and controls [57]. In this instance, MRI was also used to demonstrate impaired aortic distensibility among the cohort. Most research to date is consistent with diastolic dysfunction, or heart failure with preserved ejection fraction, being more common in CKD [58]. This concept translates to childhood hypertension when subclinical differences in systolic and diastolic function can be detected among children with BP ≥80th percentile [59].

### Association with Metabolic Risk and Other Biomarkers

Beyond hypertension, we need also consider other cardiometabolic factors that contribute to end-organ disease. Among these, adiposity and dyslipidemia are key, in-part because of their increasing prevalence including among children with CKD. In CKiD, one third of the CKiD population was overweight or obese, and BMI was as important a predictor of LVMI and LVH as those risk factors already discussed [60]. While there was no difference in prevalence by sex, there was a differential effect of BMI on LVH by sex. The OR of LVH per unit increase in BMI z-score for boys being 1.5 (95% CI 1.1 to 2.1) compared to 3.1 (95% CI 1.8 to 4.4) for girls. Odds being multiplicative, this translated to a large sex difference in LVH prevalence among obese participants (LVH observed among 34% of obese girls compared to 9% of obese boys). The Cure Glomerulonephropathy Network (CureGN) investigated the prevalence of documented dyslipidemia among 761 children with glomerulonephritis, 21% of whom were hypertensive and 51% overweight or obese [61]. Despite being a United States based study, where screening for dyslipidemia is recommended from 8 to 10 years of age for children with CKD, only half of participants were screened and among those with confirmed dyslipidemia only 9% were treated at study enrolment [62, 63]. Among those screened, the prevalence of hypercholesterolemia was 62%. This indicates there may be substantial reluctance or inertia in treating children for dyslipidemia. The authors propose this may be partly explainable by the relapsing–remitting course of some glomerular disease, but it likely also represents a missed opportunity for early intervention in some.

Oxidative stress and inflammation have been linked to long-term cardiovascular events in different populations, including youth with CKD. This association is of interest because of the potential for translation via directed interventions that might reduce oxidative stress [64, 65]. However, proving a relationship between GFR and renally excreted biomarkers is complex, as illustrated by the demonstration of a reverse association between urinary levels 8-OH deoxyguanosine and F2-isoprostane as markers of oxidative stress with eGFR and proteinuria measured annually for five years among CKiD participants [66]. A similarly extensively studied biomarker linked kidney function is uric acid, for which preclinical and observational data have failed to translate to successful clinical trials thus far [67–69].

## Interventions to Reduce BP and Modify Cardiovascular Outcomes

### Lifestyle Interventions

Lifestyle interventions have the potential to positively impact BP and cardiovascular outcomes, while simultaneously benefiting health generally and with negligible risk. There is moderate evidence supporting interventions targeting diet, weight management and exercise among children with and without CKD. More comprehensive and longer lasting programs tend to have greater impacts, although even singular counselling sessions have been associated with reductions in BP months later. One example is the Treating Resistant Hypertension Using Lifestyle Modification to Promote Health (TRIUMPH) trial for adults with drug resistant hypertension, which demonstrated a reduction in BP of –12.5 mmHg (95% CI –14.9 to –10.2) among participants who received a 4-month, comprehensive lifestyle program compared to –7.1 mmHg (–95% CI 10.4 to –3.7) among those receiving a standard, singular advice session [70]. Trials enrolling children have demonstrated similar results, noting there are relatively more data supporting family-based therapy for obesity, compared to behavioral interventions that include children or parents alone [71]. It is likely that such interventions will prove beneficial for children with CKD, but need be tailored to individual capacity and find ways to engage children who already hold a significant burden of medical care [72].

Dietary approaches center around a low-sodium (< 100 mmol/day) DASH diet. Noting that the pivotal trial enrolled adults with untreated hypertension and normal kidney function, there are data indicating that the benefits will translate to children with hypertension and CKD [73]. Among adults with hypertension and CKD, observational data indicate adherence to a DASH diet is associated with a slower progression to kidney failure [74]. Among adolescents with untreated hypertension, trial data confirmed the efficacy of DASH in reducing BP 12 months following a 6-month intervention. BP was 2.7 mmHg lower (95% CI –5.2 to –0.1) among the group receiving a specific teen-focused program compared to standard of care [75•]. Other targeted examples include the Pacific Kids DASH for Health (PacDASH) for Pacific Islander children in the upper half of the BMI distribution, which led to improvements in diet and DBP but not SBP or BMI for children in the intervention arm (delivered at 3, 6 and 9 months, observation to 15 months) [76]. Regarding the low sodium component specifically, there are plentiful data supporting a positive correlation between sodium intake and BP in children, including that these can be modified by directed intervention [77]. The only caveat to sodium reduction being the need to exclude children with a salt-losing or negative sodium-balance phenotype, which is common among children, particularly infants with CKD.

### Antihypertensive Therapy

The aforementioned ESCAPE trial demonstrated several important components to treating hypertension in children with CKD [78]. As discussed, ESACAPE embedded the importance of ABPM in clinical care, and set the treatment target of a 24-h mean blood pressure ≤50th centile. The use of ramipril as the antihypertensive agent of choice is consistent with a large body of evidence supporting specific benefit to ACEi and ARB over other agents in slowing the progression of CKD. Post-hoc analysis of ESCAPE confirm that a greater response in terms of proteinuria (initial reduction in proteinuria) predicted better preservation of kidney function [79].

The principle of intensive blood pressure control when treating hypertension in children with CKD has been further tested in the recent HOT-KID trial [80•]. HOT-KID randomized 124 children with CKD stages 2 to 5 to a treatment target of an auscultatory systolic blood pressure < 40th centile or an auscultatory systolic blood pressure between the 50th and 75th centile using ACEi or ARBs, and followed participants for a median of 39 months (IQR 28 to 52). The primary outcome, change in LVMI, was not different between groups and excluded the pre-specified clinically significant difference of 3.1 g/m^2.7^ (treatment effect -0.7 g/m^2.7^ in the intensive group, 95% CI –1.9 to 2.6). However, a significant between group difference was found for change in relative wall thickness (-0.020, 95% CI -0.039 to -0.009). It is proposed that the greater difference in Δ relative wall thickness was because this is a more sensitive and an earlier indicator of concentric remodeling. Most participants had early CKD (89% had an eGFR > 45 ml/min/1.73m^2^) and relative wall thickness was more abnormal in the groups at baseline (the mean baseline value was greater than the reference 80th percentile), compared to LVMI which was within the normal range for most participants. The study authors conclude that office BP treatment should be maintained < 50th percentile based on these results and to align with the recommendations for target ABPM.

The use of pharmacologic agents for children who do not respond fully to ACEi and ARB follows principles of treatment in other populations. The next agents used are typically long-acting calcium channel blockers and diuretics (thiazide, or loop with advanced CKD), before other antihypertensive classes such as beta blockers, alpha blockers, and direct acting vasodilators. In children with oligoanuric kidney failure on kidney replacement therapy, in whom hypertension is also related to fluid overload, it can be difficult to control blood pressure via pharmacologic means alone.

### Statins

There is limited direct evidence to support the use of statin therapy to prevent atherosclerotic events among children with hypertension and CKD. However, statins have been demonstrated to reduce the time to first atherosclerotic event among adults with CKD, regardless of blood pressure status, eGFR or dialysis status [81]. In the Study of Heart and Renal Protection (SHARP), effect size was proportional to the reduction in lipid levels. Taken together with evidence of safety and efficacy in reducing lipid levels in children with CKD, it would seem reasonable to treat children with hypertension, CKD, and dyslipidemia with statin therapy [82]. One subgroup for whom the benefits of treatment may be less certain are children with nephrotic syndrome. The strong association between nephrosis and dyslipidemia potentially outweighing the impact of statin therapy in this group, though more research is needed [83]. Early data for PCSK9 inhibitors in nephrotic syndrome appear promising and pediatric studies are awaited, following demonstration of safety and efficacy in children with familial hypercholesterolemia [84, 85].

### Emerging Evidence

For children with concurrent obesity, there is emerging evidence to support a potential role for glucagon-like peptide-1 receptor (GLP-1R) agonists. The Effect of Semaglutide Versus Placebo on the Progression of Renal Impairment in Subjects With Type 2 Diabetes and Chronic Kidney Disease (FLOW) trial was stopped in 2023 based on pre-specified efficacy criteria [86]. While FLOW excluded participants < 18 years of age, it is likely GLP-1R agonists and other weight modification medications will play some role in the future management of children with obesity that is unresponsive to lifestyle measures and causing complications such as hypertension or diabetes [87].

## Conclusion

Cardiovascular disease remains one of the most important long-term complications of childhood CKD. Hypertension is a key risk factor for cardiovascular morbidity and also linked to a more rapid progression of kidney disease. For these reasons, it is important to continue building an understanding of the relationship between hypertension and cardiovascular disease among children with CKD. Much progress has been made but the problem remains great, and there is potential for earlier and more directed interventions among at-risk children.

## Data Availability

No datasets were generated or analysed during the current study.
